# Arsenic: In Search of an Antidote to a Global Poison

**DOI:** 10.1289/ehp.113-a378

**Published:** 2005-06

**Authors:** M. Nathaniel Mead

Arsenic. No other element has such a complex and variegated past. As early as 500 B.C. the ancients knew about arsenic, whose name comes from the Greek word for *potent*. Through the centuries, this “king of poisons” was a common means of homicide. And yet, arsenic’s image has not always been so morbid. People in the Middle Ages wore arsenic amulets around their necks to ward off the bubonic plague, and women in Victorian times applied arsenic compounds to their faces to whiten their complexions. Hippocrates, the father of western medicine, recorded arsenic’s usefulness as a topical remedy for skin ulcers.

Today, arsenic compounds are still used for pharmaceutical purposes. Arsenic trioxide is known for its use in the treatment of acute promyelocytic leukemia in patients who are unresponsive to, or have relapsed from, certain chemotherapy agents. Research published in the 1 April 2005 issue of the *Journal of Clinical Oncology* suggests that arsenic trioxide may have therapeutic uses in other malignancies as well, and that it may be used in combination with other chemotherapy drugs to expand their benefits.

And yet, no toxicologist would deny that chronic arsenic exposure places people at risk for a host of adverse health effects, from skin and internal cancers (of the bladder, kidney, liver, lung, colon, uterus, prostate, and stomach) to diabetes mellitus and vascular, reproductive, developmental, and neurological effects. Studies have shown arsenic to be a potent endocrine disruptor, altering hormone-mediated cell signaling at extremely low concentrations.

Joshua Hamilton, program director of the Dartmouth College Superfund Basic Research Program, and colleagues published papers on the latter topic in the March 2001 issue of *EHP* and the August 2004 issue of *Chemical Research in Toxicology*. “We demonstrated this with the glucocorticoid receptor and subsequently showed that arsenic has similar effects on all five steroid receptors,” says Hamilton. “Furthermore, we recently found similar effects on other members of the nuclear receptor signaling family, including retinoic acid and thyroid hormone receptors.” Since these receptors are central to so many biological processes, Hamilton suggests that this may be an important way by which chronic arsenic exposure contributes to so many malignancies as well as nonmalignant diseases.

The noncancer effects of arsenic arise from both acute and chronic exposures. Among those symptoms linked with acute exposure to arsenic-laced well water (typically containing more than 1,200 micrograms per liter [μg/L]) are abdominal pain, vomiting, diarrhea, muscular weakness and cramping, pain to the extremities, erythematous skin eruptions, and swelling of the eyelids, feet, and hands. A progressive deterioration in the motor and sensory responses may also result, finally leading to shock and death.

The effects of chronic arsenic poisoning (also called arsenicosis) are more complex. Aside from cancer, these chronic effects include atherosclerosis, diabetes, hypertension, anemia, liver disorders, kidney damage, headache, confusion, peripheral neuropathy, and a variety of skin lesions, notably hyperkeratosis, or thickening of the skin, and both hypo- and hyperpigmentation.

Skin lesions are the most common outward sign of chronic arsenic exposure, though many dermatologic symptoms are thought to be mediated by nutritional factors. Studies conducted in Taiwan, India, and Bangladesh have linked high-arsenic well water with the incidence of both skin lesions and diabetes in a dose-responsive pattern. One recent population study in West Bengal, India, published in the March 2003 issue of *Epidemiology*, showed that the lowest peak arsenic ingested by a confirmed case of arsenic-induced skin lesions was 115 μg/L.

Among children, chronic arsenic exposure has also been reported to cause adverse effects on the digestive, respiratory, cardiovascular, and nervous systems. An article in the September 2004 issue of *EHP* reported intellectual impairment occurring when arsenic in drinking water exceeded 50 μg/L.

There is evidence that arsenic-exposed people who are predisposed to noncancerous skin lesions may be more vulnerable to other cancers. “During our long field experience in West Bengal and Bangladesh we observed that those who are suffering from severe keratosis appear more likely to develop cancer later on,” says Dipankar Chakraborti, director of the School of Environmental Studies at Jadavpur University in Calcutta. “Not only skin cancer but internal cancers also may arise in people who show such noncancerous lesions.”

The lung, too, seems to be a major site of action of ingested arsenic. “Lung cancer is the main cause of arsenic-related death,” says Allan Smith, director of the Arsenic Health Effects Research Program at the School of Public Health, University of California, Berkeley. “But we’re also seeing many noncancer [lung] effects, such as a tenfold [increase in the] rate of bronchiectasis in people with skin lesions in India.”

The prevalence and incidence of these noncancer manifestations of arsenic exposure is highly variable from one country to the next. For example, whereas skin pigmentation and hyperkeratosis are common indicators of arsenic exposure in Taiwan, it may be more common in India to see respiratory stress, polyneuropathy, and peripheral vascular disease linked with habitual ingestion of high-arsenic drinking water. This topic remains a very active area for epidemiologic research.

## A World Exposed

Globally, millions of people are at risk for the adverse effects of arsenic exposure. The majority of harmful arsenic exposure comes from drinking water from wells drilled through arsenic-bearing sediments. Drinking water contains primarily inorganic arsenic, which is more acutely toxic than the organic form. The other major sources of arsenic exposure are through food, soil, and air. For most people, in fact, the primary exposure to arsenic comes from food, but dietary arsenic includes primarily organic forms, which are relatively nontoxic and contribute little, if any, to the overall risk associated with exposure. (Unless otherwise indicated, all mentions of arsenic in the remainder of this article refer to the inorganic form.)

Rebecca Calderon, chief of the Epidemiology and Biomarker Branch at the Environmental Protection Agency (EPA) National Health and Environmental Effects Research Laboratory, says that preparing foods in arsenic-containing water increases the arsenic content by 10–30% for most foods, and by 200–250% for beans and grains, which absorb cooking water. Moreover, arsenic-laced irrigation water can substantially increase the arsenic content of rice and vegetables, as recently shown in several studies in Southeast Asia, including a February 2005 *Chemosphere* report on West Bengal crops and soil.

Soil- and waterborne arsenic does not readily permeate the skin, though soil can be a key source of exposure in young children who show significant hand-to-mouth activity. People are also exposed on a more sporadic basis through a hodgepodge of human activities, such as the burning of fossil fuels, waste incineration, smelting of ores, pesticide and herbicide use, coal burning, semiconductor production, and other manufacturing processes. The public health impact of these exposures is largely unknown as the epidemiologic focus has been on exposure via drinking water.

For most U.S. citizens who are on piped water systems, drinking water is not a major source for arsenic exposure. Nonetheless, in certain areas in the West, Midwest, Southwest, and Northeast, people drinking well water may be exposed to arsenic levels ranging from 50 to 90 μg/L, well above the EPA’s guideline of 10 μg/L. To date, no statistically significant relationships have been found between arsenic exposure and cancer in these areas.

The situation in Bangladesh and West Bengal is radically different: arsenic exposure through drinking naturally contaminated groundwater is widespread and often excessive. This situation began in the 1970s, when the United Nations Children’s Fund, in response to epidemics of cholera, dysentery, and other waterborne infectious diseases, spearheaded an effort to switch the region’s population from drinking surface waters to groundwater. Millions of tube-wells were drilled into arsenic-rich sediments; as a result, in many of these wells arsenic levels reach 500–1,000 μg/L and even higher.

Field studies have shown that many people living in a vast geological zone known as the Ganga-Meghna-Brahmaputra plain are being exposed to high arsenic levels in the water. A large portion of this plain, an area totaling 500,000 square kilometers and spanning all of Bangladesh and most of India, shows significant groundwater arsenic contamination, putting more than 500 million people at risk of chronic arsenic poisoning, says Chakraborti. He published these alarming estimates in the June 2004 issue of the *Journal of Environment Monitoring*. With 80% of Bangladeshis estimated to be at risk of arsenic-related diseases, the World Health Organization (WHO) has labeled this “the worst mass poisoning in history.”

Large areas of China also face severe arsenic exposure from groundwater contamination, with more than 3 million people affected, based on estimates in the August 2004 issue of *Toxicology and Applied Pharmacology*. In Shanxi Province alone, an estimated 900,000 people are at risk of arsenicosis. Among the investigated villages in Shanxi, an average of 52% of wells give water containing arsenic concentrations higher than 50 μg/L, according to a recent report from the School of Public Health at China Medical University in Shenyang.

A unique type of exposure, resulting from the burning of arsenic-rich coal, is found in Guizhou Province, an area of endemic arsenicosis. Guizhou inhabitants commonly use this coal for cooking, heating, and drying their dietary staples of corn and hot peppers. The coal is burned in open stoves without chimneys, resulting in contamination of both the indoor air and the foods being prepared. At this time, arsenicosis is known to affect eight provinces, but most of China has not been studied, and new endemic areas are continuously emerging. Reports on arsenicosis in China actually preceded those from Bangladesh and India, but have been overlooked due to limited scientific exchange and publication.

Other countries with arsenic-rich groundwaters include Argentina, Chile, Mexico, Cambodia, Vietnam, Thailand, Nepal, and Ghana. In the Obuasi area of Ghana, arsenic contamination of food and water has been linked with gold-mining activities. Much of the gold in the Obuasi mines is locked in pyrite and arsenopyrite, both associated with arsenic and sulfur. The extraction of the gold results in the release of airborne particles that include large concentrations of arsenic. At least 10% of Ghana’s rural borehole wells have arsenic concentrations exceeding 10 μg/L. In the Terai region of Nepal, inhabited by half the country’s total population, hundreds of thousands of shallow tubewells have been installed by various agencies, and groundwater is the primary source of drinking water. According to Chakraborti, around 500,000 people in Terai are at risk of arsenic poisoning from drinking this water, and up to 1 in 20 people may show skin lesions indicative of arsenicosis.

## The Arsenic–Cancer Equation

Today, researchers around the world are racing against the clock to unravel the secrets of arsenic’s workings, including how it influences the cancer process and thereby increases cancer risk. Although inorganic arsenic is generally held to be more acutely toxic, some researchers argue that the organic metabolites of arsenic may be the ultimate carcinogens. One of these metabolites, DMA, has been shown in rodents to induce bladder cancer and to promote tumor growth in several other organs. A review article focusing on induced disturbances of calcium homeostasis, genomic damage, and apoptotic cell death caused by arsenic and its organic metabolites appears in the June 2005 issue of *EHP*.

There is general agreement that arsenic does not directly interact with DNA, and that its toxic effects occur through indirect alteration of gene expression, such as via the perturbation of DNA methylation, inhibition of DNA repair, oxidative stress, and altered modulation of signal transduction pathways. Many of these mechanisms are overlapping, interdependent, and heavily influenced by factors in the cellular environment. For example, arsenic promotes both oxidative stress and impaired DNA repair, and yet both of these effects tend to amplify mutation rates, thus increasing the likelihood of cancer.

Another indirect mechanism is the influence of growth-stimulating chemicals or cytokines generated in response to arsenic exposure. Dori Germolec, a research scientist at the NIEHS Laboratory of Molecular Toxicology, has been approaching the arsenic question from the standpoint of cytokine biology. “Arsenic alters the production of inflammatory cytokines and does so persistently over time,” Germolec says. “These effects on cytokines seem to relate to its effects on the skin. Arsenic seems to stimulate progenitor cells that could ultimately be responsible for tumor formation. This is just one of a number of mechanisms that has biological plausibility.” Research published in the April 2004 issue of *EHP* by Toby Rossman, an environmental science professor and program director of the Molecular and Genetic Toxicology Program at New York University, has demonstrated similar relationships in animal models as well as in cultured human cells.

Studies of differences in arsenic metabolism between individuals have led to further insights—and further questions. The importance of individual arsenic metabolites in terms of cancer induction is still being determined. All of the human populations studied thus far have been found to methylate inorganic arsenic, but the patterns of arsenic metabolites in urine show substantial interindividual variation. Within any given population, individuals differ in the quantity and distribution of the various metabolites of arsenic excreted by the kidney. If some happen to excrete more of the carcinogenic metabolites or are unable to metabolize arsenic efficiently, they may be more vulnerable to cancer. This variation may be affected by a variety of factors, including dose level, route(s) of exposure, diet, and the particular type of arsenic to which the individual is exposed. Polymorphisms in genes that code for the enzymes important in metabolism, such as arsenic methyltransferase, have also been implicated as accounting for some of this variability.

No one yet knows how this interindividual variation in arsenic metabolism actually affects cancer risk. “This is a difficult question since when you deal with the carcinogenicity of inorganic arsenic you are dealing with six or more distinct [metabolites],” says H. Vasken Aposhian, a molecular and cell biology professor at the University of Arizona. Aposhian is involved in studies in New England, Mongolia, Romania, Mexico, and Kazakhstan to identify unique or abnormal arsenic urine profiles in people who develop cancer in areas of high arsenic exposure. Once studies reveal which of these metabolites are promoters and/or carcinogens, it will be possible to better answer the riddle of interindividual variation in vulnerability to arsenic-induced effects.

The metabolite MMAIII presently is one of the leading candidates as a potential cancer inducer. If MMAIII turns out to be carcinogenic, an increased or decreased amount in the urine might prove useful as a marker for potential future arsenic-mediated cancer.

In time, the identification of reliable exposure markers could help identify groups that may be more susceptible to cancer at the levels of arsenic exposure typically found in the United States (less than 50 μg/L). At the present time, the carcinogenic risk of such exposures is unclear. Biomarkers would provide a more detailed picture of individual arsenic exposure and how the body is responding to that exposure. “The low-dose extrapolations used for risk assessment purposes may be subject to error in part because they are based more on ecologic data than on individual measures of exposure,” says Margaret Karagas, an epidemiology professor at Dartmouth College. “Use of relevant markers in human tissue samples eventually may help us sort out the risk at lower levels of exposure.”

One practical biological marker identified by Karagas is toenail clippings, with arsenic content measured via instrumental neutron activation analysis. Using this measure, she and her colleagues reported in the June 2004 issue of *Cancer Causes & Control* on a case–control study in New Hampshire suggesting an increased cancer risk associated with moderate arsenic exposure, but only in smokers.

## An Emerging Consensus: Arsenic Does Not Act Alone

Studies such as Karagas’s point to the growing recognition that arsenic does not always operate alone. Rather, arsenic appears to work with other factors to promote cancer, at least at some target sites. “Animal models indicate it takes a promoter or some genotoxic carcinogen to get arsenic to produce skin cancers,” says Michael Waalkes, section chief of the Inorganic Carcinogenesis Section at the National Cancer Institute Laboratory of Comparative Carcinogenesis, housed at the NIEHS. “When you always see this kind of cotreatment effect, it makes it harder to nail down the precise contribution of arsenic to the final tumor.”

The classic cofactor in this regard may be tobacco smoke. “There is mounting evidence of a malignant synergy between smoking and arsenic,” says Smith. “Smokers are at an increased risk from arsenic in drinking water and appear to comprise a susceptible subpopulation.” A study by Smith and colleagues, published in the November 2000 issue of *Epidemiology*, found that the relative risk of lung cancer for Chileans who smoked and had high arsenic in their water was 32 times that of nonsmokers with low arsenic concentrations in their water. In contrast, the lung cancer risk of smokers without arsenic in their water was about 6 times that of nonsmokers. Similar findings have come from studies in Taiwan and New Hampshire.

Other cofactors are also gaining attention. Rossman’s group was among the first to hypothesize that arsenic requires a carcinogenic partner—in their April 2004 *EHP* article and another in the 1 August 2004 issue of *Toxicology and Applied Pharmacology*, they reported finding that arsenic plus ultraviolet (UV) radiation exposure led to a dose-related increase in skin cancers in mice compared with mice exposed to UV light alone. The tumors in mice treated with arsenite plus UV light also appeared earlier and were larger and more invasive than those in mice exposed to UV light alone. At the 2004 Third International Conference on Comparative Physiology and Biochemistry, Rossman reported that selenium deficiency also enhanced the carcinogenic effects of arsenic.

Such insights may carry over to the epidemiological realm. In Bangladesh and West Bengal, for example, the most likely cofactors for arsenicosis include malnutrition (with resulting deficiency of selenium and other nutrients that can affect arsenic metabolism) and agricultural activities that lead to frequent sun exposure. Not only does selenium seem to help protect against the toxic effects of chronic arsenic exposure, but high levels of chronic arsenic ingestion from well water may accelerate the excretion of selenium, according to research published in the 5 May 2004 issue of *Science of the Total Environment*.

“We need to find out whether Bangladesh and other poverty-stricken countries with arsenic-tainted groundwater may benefit by this relatively cheap strategy of supplementing the diet with selenium,” says Floyd Frost, an epidemiologist at the Lovelace Respiratory Research Institute in Albuquerque. “We need solutions that are cheap and doable. If you’re in Bangladesh, there just isn’t much money for expensive mitigation strategies.”

However, Smith notes that he and colleagues found only modest increased risks in West Bengal with some dietary deficiencies. He and others contend that the top priority should be to reduce arsenic exposure. Other approaches being explored include rainwater collection, novel filtration systems, chelation, and deep community wells, as well as the use of antioxidants, methionine (an amino acid), and other dietary supplements that may limit arsenic’s toxicity. [For more information on remediation strategies, see “Columbia Center Digs Deeper into Arsenic Dilemma” and “Metal Attraction: An Ironclad Solution to Arsenic Contamination?” p. A374 and A398 this issue.]

## A Special Population: The Very Young

Infants and children are deemed to be more susceptible than adults to the adverse effects of arsenic and other toxic substances. Chakraborti has observed that arsenical skin lesions show up sooner in children than they do in adults. If the child’s nutrition is poor, outward signs of arsenic toxicity manifest even sooner and at less extreme levels of exposure. An additional concern is the potential for increased sensitivity of children to arsenic-associated neuropsychological effects such as reduced verbal IQ scores, as reported in the September 2004 issue of *EHP*.

Chakraborti speculates that infants and children may be intrinsically more susceptible than adults due to differences in metabolism, a view supported by some preliminary studies. “In one of our studies on an arsenic-affected population in Bangladesh, we found that the second step in arsenic metabolic pathways is more active in exposed children in comparison with exposed adults,” he says. In the June 2005 issue of *EHP*, Maria Mercedes Meza and colleagues identified a developmentally restricted component of arsenic metabolism, a genetic association with urinary arsenic metabolites that applied only to children.

Complicating this scenario is the special threat posed by *in utero* exposure to arsenic. One of the concerns here is that low-level exposures may have a greater impact if experienced *in utero* than if experienced in childhood or adulthood. Waalkes and his colleagues were the first to identify the transplacental carcinogenic potential of arsenic. They duplicated this finding in several rodent studies, reported in the 1 August 2004 issue of *Toxicology and Applied Pharmacology* and the 20 May 2004 issue of *Toxicology*.

“The critical window of exposure for mice equates to about the middle three months of pregnancy in humans,” says Waalkes. “This could lead to a fifty percent increase in the risk of hepatocellular carcinoma for adults. This is a reproducible phenomenon, and it has alarming implications for *in utero* exposures in humans.” The first half of fetal development is a period of very high sensitivity because of a high rate of cell proliferation, cell differentiation, and gene imprinting, all of which, when disrupted, can lead to carcinogenesis.

Smith’s studies of bladder and lung cancers also have indicated that there may be a long latency—40 years or more for these cancers—from arsenic exposure to the manifestation of malignant disease. For example, he has found very high lung cancer risks in Chilean adults who were exposed as children or *in utero*. He notes that it is critically important to study large populations with significant and well-documented arsenic exposure. Smith says Chile has the best-documented exposure in the world.

“In any country where people are exposed to high levels of arsenic, if nothing else is done, they should focus on protecting pregnant women, providing them with low-arsenic water,” says Waalkes. “That would be my top priority if I could advise the governments of those countries on what to do.”

## How Much Protection Is Enough?

Although the effects of severe arsenic contamination are well established, there is much debate about the risk associated with chronic ingestion of drinking water that contains arsenic levels lower than regulatory standards. The WHO adopted a standard of 10 μg/L in 1993. Bangladesh and many other developing countries use a guideline of 50 μg/L. Beginning in January 2006 the maximum contaminant level for inorganic arsenic permitted in U.S. drinking water will be 10 μg/L, although scientists still debate this standard.

Part of the uncertainty regarding the 10 μg/L standard stems from the absence of epidemiologic data to help determine the exact shape of the dose–response curve, particularly at exposures under 10 μg/L. Cancer risks at these levels of exposure may be about 1 in 300 people, according to the National Research Council report *Arsenic in Drinking Water: 2001 Update*. However, says Smith, epidemiology will never prove such risks are real. He points to the fact that large numbers of studies throughout the world were required to eventually demonstrate that nonsmokers married to smokers had an increased risk of lung cancer, even though such risk involves about 1 in 100 persons.

Still, some argue that different study designs and larger sampling will, in time, provide adequate data to answer the question of whether there is a level of arsenic exposure below which health effects do not develop. In the interim, the precautionary principle holds sway; policy makers assume that the burden of proof for potentially harmful actions or policies rests on the assurance of safety, and that when there are threats of cancer or other serious diseases, scientific uncertainty must be resolved in favor of prevention.

Acceptance of the limitations of epidemiologic research in detecting the risk associated with low-level exposures lies at the very heart of this principle. “It is possible that the effects may be nonlinear, with certain extremely low levels of arsenic exposure posing no excess risk,” says Karagas. “In epidemiologic studies, however, it is important to distinguish between ‘no effect’ and ‘inability to detect an effect’ due to various methodological limitations.”

There is also, she says, a critical need for further data on other health outcomes and in potentially susceptible subgroups such as pregnant women and children, and those particularly at risk due to genetic or lifestyle factors. By studying the whole population but not susceptible subgroups, scientists may be missing key pieces to the arsenic puzzle.

Hamilton concurs but emphasizes a more mechanism-based rationale. He theorizes that arsenic at different doses may act by different mechanisms, perhaps producing different patterns of disease. For example, the patterns of disease in areas such as Bangladesh that have high and endemic arsenic contamination may be quite different than the patterns seen at the lower doses encountered elsewhere. “We have observed an almost completely nonoverlapping pattern of gene expression changes with a low versus a high dose of arsenic, almost as if they were two different agents,” says Hamilton.

“At the lower, noncytotoxic dose,” he explains, “we saw an approximately equal number of genes that were increased as were decreased, whereas at the higher, cytotoxic dose, virtually all of the significant changes involved activation of genes.” Most of the genes in the latter case were members of stress response and apoptosis pathways. Taken together with Hamilton’s studies of the endocrine-disrupting effects of low to moderate arsenic levels, this indicates the importance of examining arsenic at doses that are directly relevant to the end point of interest.

## On the Threshold of a New Understanding

A major challenge for future research is the issue of linking genetic polymorphisms with arsenic-related disease susceptibility. “Since arsenic metabolism seems to be a key to the carcinogenic process, sorting out these polymorphisms will be important, but this is extremely difficult to do,” says Julian Preston, director of the EPA’s Environmental Carcinogenesis Division and a member of the committee that produced *Arsenic in Drinking Water: 2001 Update*. “You need to see a very strong association between a particular polymorphism and the cancer end point in order to establish a link.” To date, a few polymorphisms have been identified in an indigenous population in Chile that may confer protection against the carcinogenic effects of arsenic exposure, but the findings are only suggestive.

Given that humans appear to be substantially more sensitive than experimental animals to arsenic-induced cancers, more epidemiologic research will be needed to assess the effects of early-life exposures for child as well as adulthood cancers. “Humans remain the most sensitive species when it comes to understanding the toxicity of arsenic,” says Calderon. “Despite several attempts to use rodents and other animal species, those assays and experiments have had limited success in explaining what appears to be a rather unique response on the part of *Homo sapiens* to arsenic. This represents a unique challenge, and perhaps the keys reside in emerging areas of genomics, proteomics, or molecular epidemiology.” Childhood exposure to arsenic has emerged as a potential regulatory concern.

Arsenic contamination of drinking water is among the most awesome environmental health challenges of our time. With hundreds of millions of people affected in Southeast Asia and elsewhere, the need for effective arsenic mitigation strategies has never been greater. Thus the focus is moving beyond exposure to include those physiologic variables that may mediate the effects of exposure and that correlate with adverse effects in humans.

Exposures associated with arsenic due to cooking and agricultural activities (including herbicide and pesticide use) should be explored, along with the identification and control of other carcinogenic compounds that may act as cocarcinogens. Such efforts could, in time, result in profound public health benefits and alleviate a great deal of suffering.

For people living in areas where arsenic exposure is less extreme, the question of whether arsenic is safe below a certain dosage level remains central. Many scientists assert that only biological data based on measurements of the variation in human metabolic responses to arsenic will resolve the low-dose controversy. Such data will pave the way for developing biologically based dose–response models that should greatly enhance our understanding of arsenic’s carcinogenic potential. Only with persistent inquiry and innovative investigation will the elemental mystery of arsenic be solved.

## Figures and Tables

**Figure:**
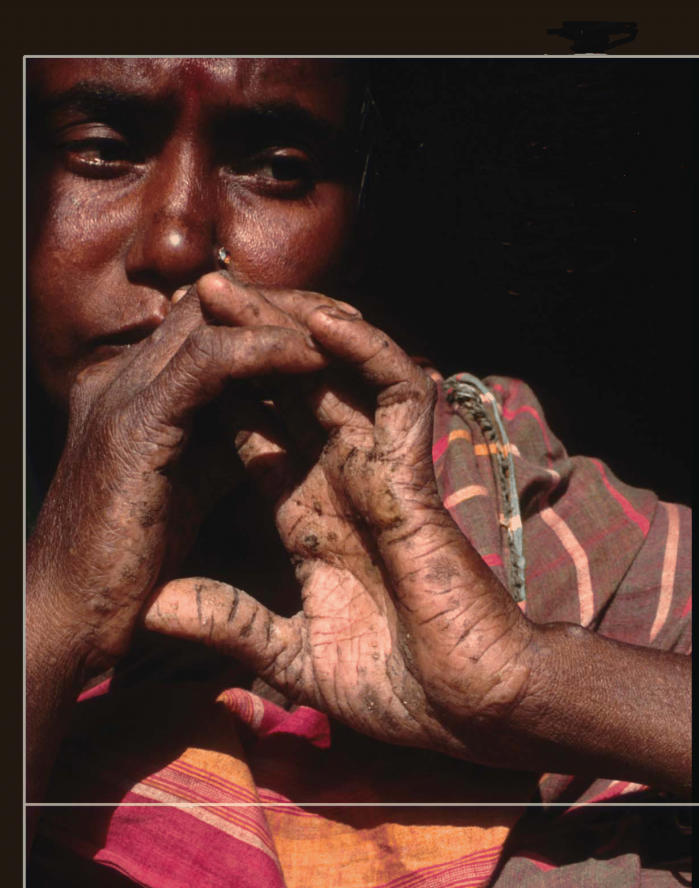


**Figure:**
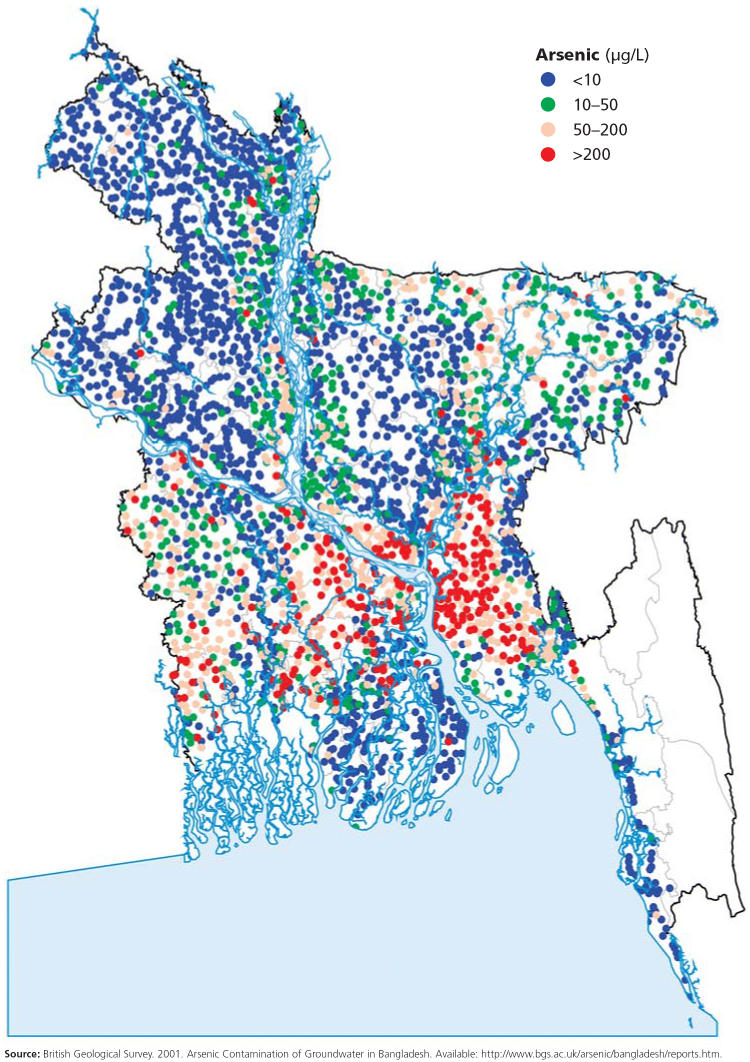
Arsenic concentrations in Bangladeshi tubewells

**Figure:**
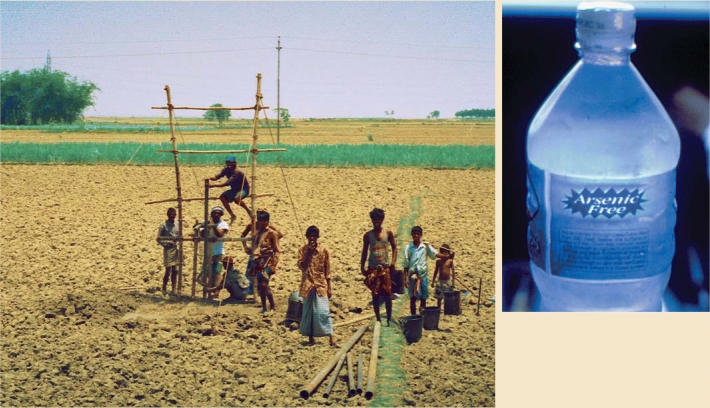
**Good intentions gone awry.** Villagers drill a tubewell in Bangladesh (left). Encouraged as a solution to pathogenic contamination of surface waters, such wells have resulted in exposure of millions to arsenic, leading to the need for alternative water sources (above).

**Figure:**
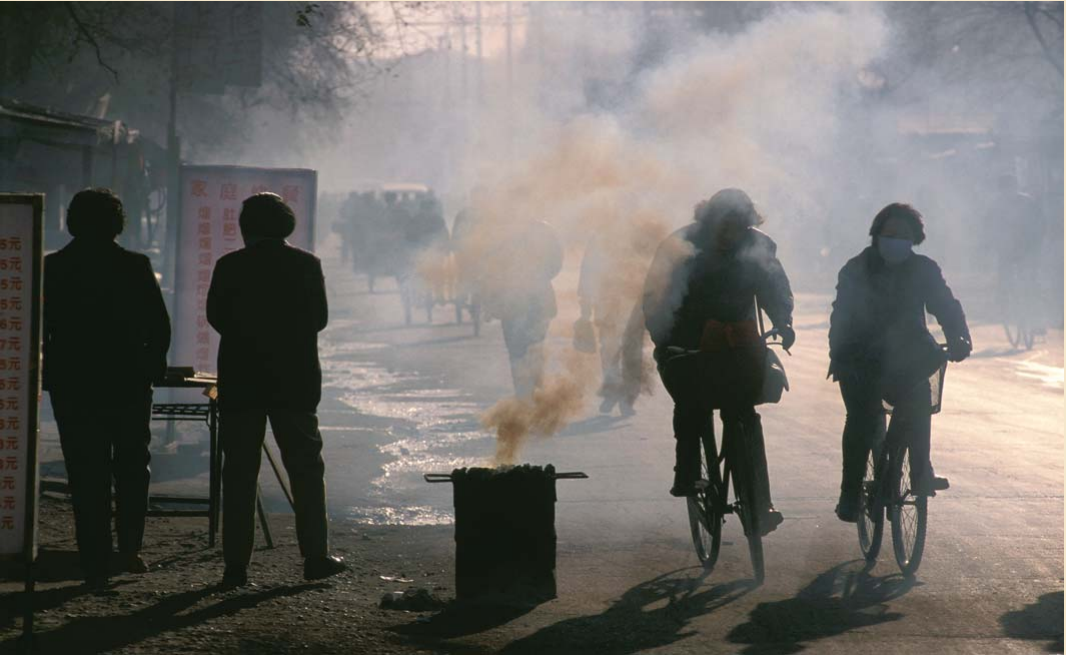
**Coal catastrophe.** Cyclists on their way to work in Guizhou Province, China, pass through smoke pouring out of a coal-burning cooking stove. Exposure to the arsenic-rich coal burned in this region has resulted in endemic arsenicosis.

**Figure:**
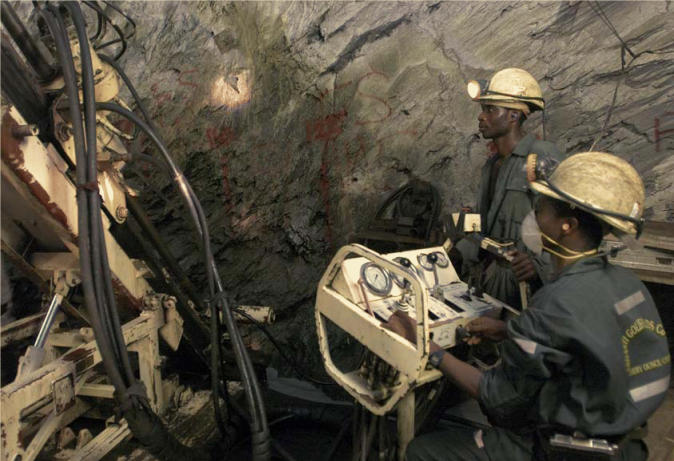
**Fool’s gold?** Gold mining in areas of Ghana such as the Ashanti Goldfields in Obuasi results in the release of airborne arsenic particles that also have been linked to food and water contamination.

**Figure:**
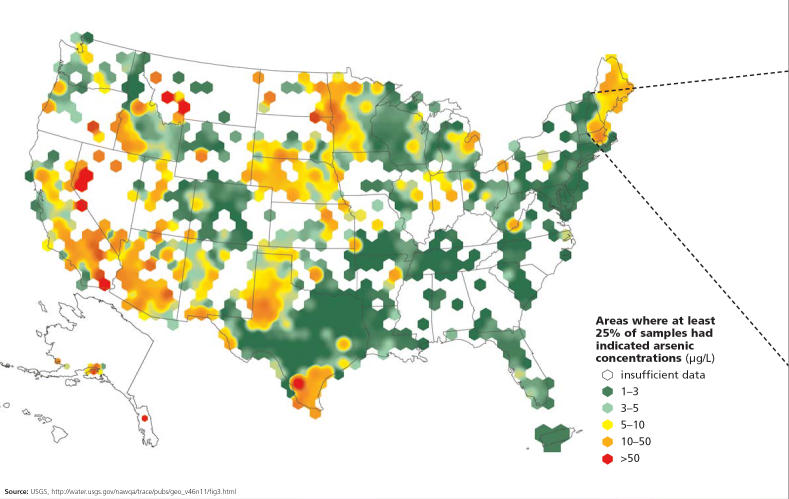
Arsenic concentrations across the United States . . .

**Figure:**
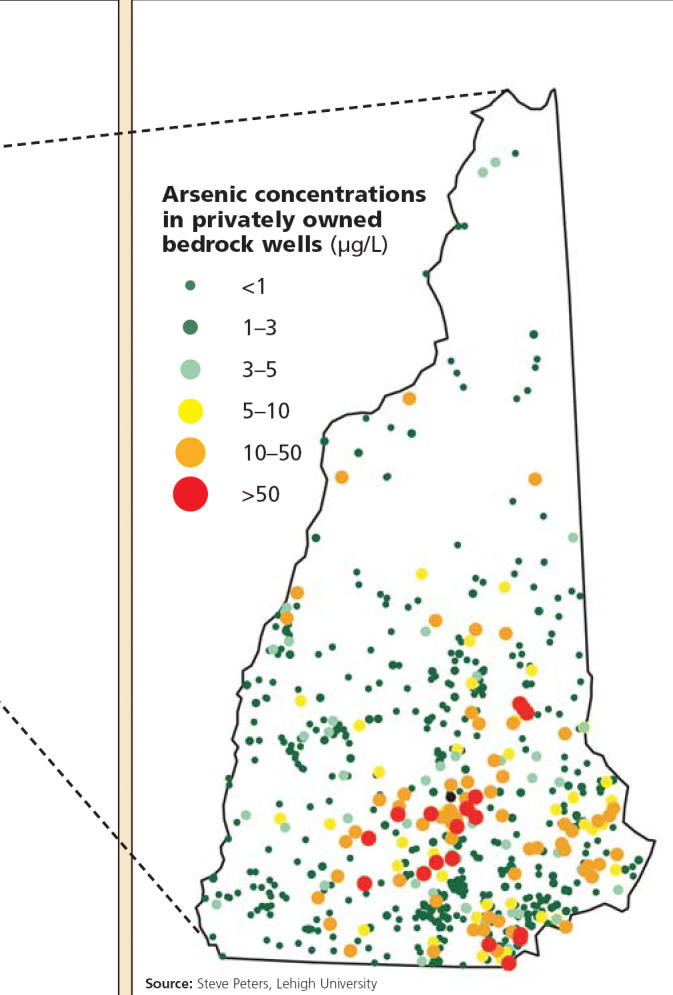
. . . and in New Hampshire

**Figure:**
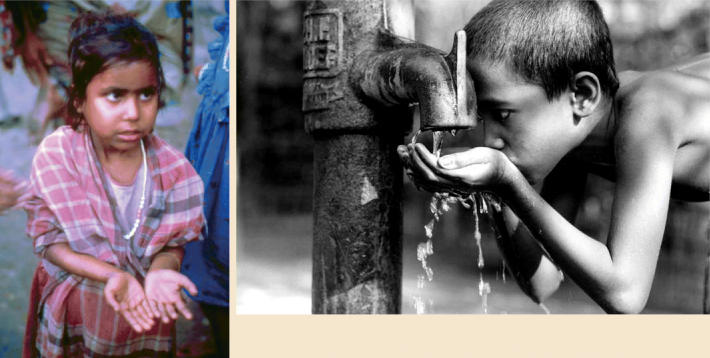
**Special victims.** New information indicates that children metabolize arsenic differently than adults, and provides compelling reason to further study the effects of the element in vulnerable populations.

